# Enhanced Amelioration of High-Fat Diet-Induced Fatty Liver by Docosahexaenoic Acid and Lysine Supplementations

**DOI:** 10.1155/2014/310981

**Published:** 2014-05-25

**Authors:** Hsin-Yu Lin, Chih-Chien Chen, Yu-Jen Chen, Yuan-Yu Lin, Harry J. Mersmann, Shih-Torng Ding

**Affiliations:** ^1^Department of Animal Science and Technology, National Taiwan University, No. 50, Lane 155, Section 3, Keelung Road, Da'an District, Taipei City 106, Taiwan; ^2^Institute of Biotechnology, National Taiwan University, No. 81, Changxing Street, Da'an District, Taipei City 106, Taiwan

## Abstract

Fatty liver disease is the most common pathological condition in the liver. Here, we generated high-fat diet-(HFD-) induced nonalcoholic fatty liver disease (NAFLD) in mice and tested the effects of docosahexaenoic acid (DHA) and lysine during a four-week regular chow (RC)feeding. Our results showed that 1% lysine and the combination of 1% lysine + 1% DHA reduced body weight. Moreover, serum triglyceride levels were reduced by 1% DHA and 1% lysine, whereas serum alanine transaminase activity was reduced by 1% DHA and 1% DHA + 0.5% lysine. Switching to RC reduced hepatic lipid droplet accumulation, which was further reduced by the addition of DHA or lysine. Furthermore, the mRNA expressions of hepatic proinflammatory cytokines were suppressed by DHA and combinations of DHA + lysine, whereas the mRNA for the lipogenic gene, acetyl-CoA carboxylase 1 (ACC1), was suppressed by DHA. In the gonadal adipose tissues, combinations of DHA and lysine inhibited mRNA expression of lipid metabolism-associated genes, including ACC1, fatty acid synthase, lipoprotein lipase, and perilipin. In conclusion, the present study demonstrated that, in conjunction with RC-induced benefits, supplementation with DHA or lysine further ameliorated the high-fat diet-induced NAFLD and provided an alternative strategy to treat, and potentially prevent, NAFLD.

## 1. Introduction

Obesity, in response to excessive nutrients and energy intake, and inadequate energy expenditure, is recognized as a low-grade and chronic inflammatory state, which leads to the metabolic syndrome, including insulin resistance, type II diabetes, atherosclerosis, hypertension, and fatty liver disease (FLD) [1, 2]. These pathologies are seriously threatening the health of the world populations and knowledge of the underlying mechanisms will provide insights into the future development of preventive and therapeutic strategies.

FLD, also known as hepatosteatosis, is a reversible condition characterized by massive accumulation of triglyceride in the liver cells, due to energy overload and impaired hepatic lipid metabolism [3]. Obesity-associated low-grade and chronic inflammatory response arises from free fatty acid-induced production of proinflammatory cytokines, including TNF-*α*, IL-1, and IL-6, by metabolic tissues, such as the liver, muscle, and adipose tissue, and by infiltrated immune cells [4]. This impairs insulin sensitivity and metabolic homeostasis, leading to increased hepatic gluconeogenesis and lipogenesis, reduced lipolysis, and reduced muscle glucose uptake and adipose lipolysis [5, 6]. When not properly controlled, FLD may progress toward steatohepatitis or liver cell death, ultimately leading to cirrhosis, liver failure, and hepatocellular carcinoma [3].

The increasing insulin sensitivity is the major approach to treat nonalcoholic FLD (NAFLD) in humans by means of weight loss, exercise, and use of various drugs [7, 8]. Additionally, nutrients such as docosahexaenoic acid (DHA), a* n*-3 polyunsaturated fatty acid, have beneficial effects in reducing obesity and the metabolic syndrome [9] and reducing the occurrence of NAFLD induced by trans-10, cis-12-conjugated linoleic acid [10, 11]. In terms of liver-associated conditions, consumption of* n*-3 PUFA reduces serum alanine transaminase (ALT) levels, a marker of liver injury due to excessive triglyceride accumulation, in NAFLD patients [12]. In the study of Arai et al. [13], DHA-enriched fish oil feeding lowers plasma levels of insulin, triglyceride and total cholesterol and liver triglycerides, and fat droplet accumulation compared to the lard/safflower oil-fed group. Also, supplementation of DHA to cultured hepatocytes diminishes hydrogen peroxide-induced oxidative stress and DNA damage, an early event in the initiation and progression of liver injury [14]. As an* in vitro* NAFLD model, Tai et al. [15] treated oleic acid-induced SK-HEP-1 cells with DHA and found that the intracellular triglyceride levels are decreased through the reduction of the expression of lipogenic genes, such as sterol regulatory element-binding protein 1c (SREBP-1c), fatty acid synthase (FAS), and acetyl-CoA carboxylase 1(ACC1).

Lysine, an essential amino acid, is important for proper growth and plays a fundamental role in the production of carnitine, a nutrient which shuttles long-chain fatty acids into mitochondria for energy production and assists in lowering the cholesterol levels [16]. In the late 1950s [17], it was shown that lysine supplementation, with corn as the sole protein source, prevents fatty liver in rats. Upon feeding a lysine/threonine-deficient diet, rats exhibit lowered levels of 20 : 5, 22 : 5, and 22 : 6 fatty acids in hepatic total lipids, suggesting that lysine is associated with the metabolism of polyunsaturated fatty acids (PUFA) [18]. Furthermore, rats fed a diet containing 5% L-lysine exhibit lower serum ALT and aspartate transaminase activities, and this supplementation also effectively protects the rats from the hepatotoxic effects of D-galactosamine [19].

Given the above-mentioned beneficial effects of DHA and lysine on NAFLD, and the fact that reducing energy intake and increasing physical activities to induce weight loss are generally recommended for patients diagnosed with NAFLD, we propose that supplementation of a regular chow diet (RC; a low-fat diet) with DHA and lysine may have a synergistic effect in improving the fatty liver condition. Both* in vivo* and* in vitro* experiments in high-fat diet- (HFD-) fed mice and human hepatic carcinoma SK-HEP-1 cells, respectively, were used to test this hypothesis and suggest an approach to treat NAFLD and elucidate the underlying mechanisms.

## 2. Materials and Methods

### 2.1. Animals and Diets

Male C57BL/6 mice (4 weeks of age) were purchased from the National Taiwan University Animal Center and the animal protocol was approved by the Animal Care and Use Committee of National Taiwan University. Mice were fed a high-fat diet (HFD; 35.5% fat; Bioserve F3282, Frenchtown, NJ, USA) for 23 weeks to induce the fatty liver symptom. Body weight was measured every week and blood samples were collected every month. To verify fatty liver induction by histological analysis (see below), two mice were randomly sacrificed from each group after 8, 12, and 16 weeks of feeding. After 23 weeks of HFD feeding, mice were fed regular chow (RC; 4.5% fat; Labdiet 5001, St. Louis, MO, USA) supplemented with different combinations of DHA and lysine, including control (saline), 0.5 and 1% (based on average daily dietary intake) lysine, 1% DHA, 0.5% lysine + 1% DHA, and 1% lysine +1% DHA (*n* = 8 in each group). Mice were treated with each nutrient supplement daily by gavage [20] for four weeks, during which time the mice were weighed every four days. At the end of the experiment, mice were sacrificed by exposure to carbon dioxide and the liver, gonadal white adipose tissues, and blood samples (from the cheek) were collected. Portions of the livers and white adipose tissues were fixed in formalin for histochemical staining and other portions were snap-frozen in liquid nitrogen and stored at −80°C for future analysis.

### 2.2. Measurement of Serum Triglyceride and Alanine Transaminase

Blood samples were centrifuged for 15 min at 2000 ×g and the serum samples were stored at −80°C for future analysis. Triglyceride (TG, kit TR 213 from Randox Laboratories, Antrim, UK) and ALT (AL 1268, Randox Laboratories) were colorimetrically measured according to the manufacturer's instructions. Samples were measured in duplicate and the average was the datum.

### 2.3. Histological Analysis

Liver tissue was excised from the bottom of the left lobe, fixed in 10% formalin, and embedded in paraffin. Sections of 4 *μ*m thickness were sliced and stained with hematoxylin/eosin according to standard procedures.

### 2.4. Image Analysis

The area of lipid droplets in the liver sections was quantified by the ImageJ software (NIH, Bethesda, MD, USA), which utilizes the color and shape of the images to determine the percentage of lipid droplets in the whole field.

### 2.5. Cell Culture

Human hepatic carcinoma SK-HEP-1 cells obtained from ATCC were cultured (to ~80% of confluence in a 6 cm dish) in Dulbecco's Modified Eagle Medium (DMEM) (Invitrogen, Carlsbad, CA, USA) supplemented with 10% fetal bovine serum (Biological industries, Beit Haemek, Israel) at 37°C in an atmosphere of 5% CO_2_ in air. To induce fat accumulation (as confirmed by oil red O staining), SK-HEP-1 cells were treated with serum-free medium containing 1 mM oleic acid [10] for 48 hours, followed by 0.1 mM DHA (Cayman Chemical, Ann Arbor, Michigan, USA) and 4 mM lysine (dissolved in deionized water) treatments in serum-free medium containing 1% bovine serum albumin (United States Biological, Swampscott, MA, USA) for 24 hours as previously described [15].

### 2.6. Quantitative Reverse Transcription-PCR

To quantify the expression levels of genes associated with hepatic lipid metabolism and inflammation, total RNA was extracted from tissues and cells using TRIzol (Invitrogen, Carlsbad, CA, USA), digested with DNase I (Ambion, Austin, TX 78744, USA) to remove the contamination of genomic DNA, and transcribed to cDNA by High Capacity cDNA Reverse Transcription kit (Applied Biosystems, Foster City, CA, USA). Real-time quantitative PCR reactions were performed on CFX96 Real-Time PCR Detection System (Bio-Rad, Richmond, CA, USA) using a DyNAmo Flash SYBR Green Kit (Finnzymes, Espoo, Finland). Running conditions for real-time PCR were initial denaturation at 95°C for 7 min and denaturation at 95°C for 10 s, followed by annealing/extension at 60°C for 30 s for a total of 39 cycles. The primer sequences were listed in Table 1. Threshold cycle (Ct) values were obtained and relative gene expression was calculated using the formula (1/2)^Ct  target  gene−Ct  *β*-actin^ [21]. Values were normalized to *β*-actin levels in the same sample and all measurements were performed in triplicate.

### 2.7. Statistical Analysis

Statistical significance among different experimental groups was determined by one-way analysis of variance (ANOVA) and Tukey's test was used to evaluate the differences among the means of different treatments. Results were expressed as mean ± SEM. *P* values ≤ 0.05 were considered statistically significant.

## 3. Results

### 3.1. Induction of NAFLD in Mice by HFD

The HFD induced higher body weight gain (up to ~110% for HFD versus ~55% for RC; see Figure S1A  in Supplementary Material available online at http://dx.doi.org/10.1155/2014//310981), massive lipid droplet accumulation in the liver (Figure S1B), and profound hypertrophy of gonadal adipocytes (Figure S1C) during the 24-week treatment period.

### 3.2. RC and Combinations of DHA and Lysine Supplementations Reduced the Body Weight  of HFD-Fed Mice

**Figure 1:**
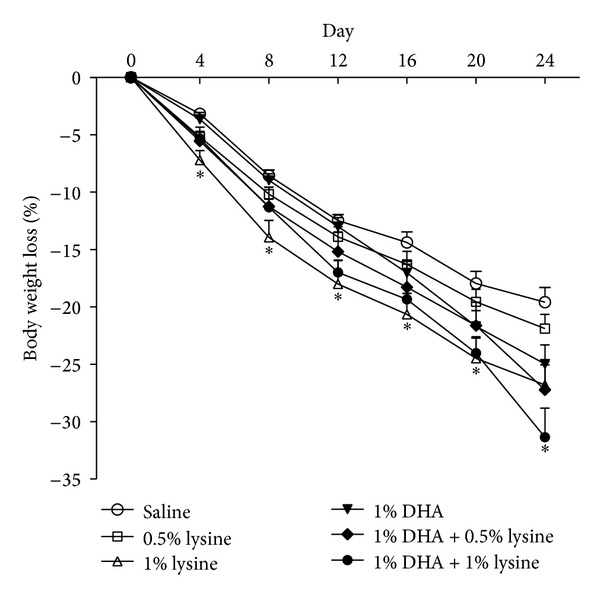
Effects of lysine and DHA supplementations on body weights of HFD-fed mice switched to RC. After 23 weeks of HFD feeding, mice were switched to RC (a low-fat diet on day 0) supplemented with combinations of lysine and DHA as indicated for 24 days. The body weight was measured every 4 days. Values are expressed as means ± SEM (*n* = 8). *indicates a treatment effect (*P* < 0.05).

After 23 weeks of HFD feeding and fatty liver induction, body weight was reduced upon RC feeding and DHA and lysine supplementations (Figure 1). As expected, switching to RC, which contains only 4.5% of fat, led to progressive reduction in body weight. However, except at day 24, a somewhat unexpected further reduction, beginning at day four, was observed for the 1% lysine group compared to RC alone control, indicating a beneficial effect of lysine on obesity. The 1% DHA supplementation groups (with or without lysine), at day 24, reduced body weight compared with the RC group, confirming the antiobesity effect of DHA.

### 3.3. DHA and Lysine Supplemented-RC Feeding Decreased Serum TG and ALT Levels of HFD-Induced Fatty Liver Mice

**Figure 2:**
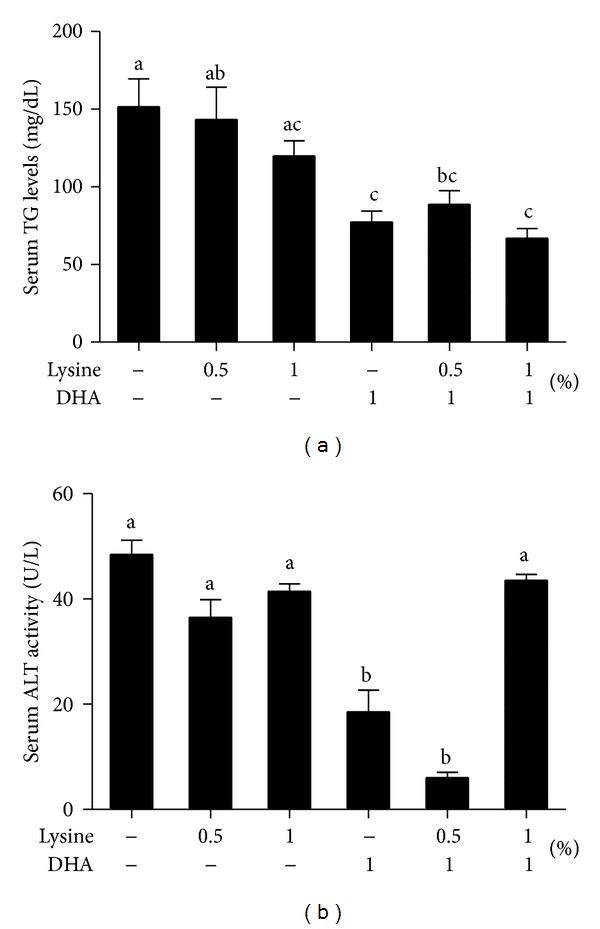
Effects of lysine and DHA supplementation on serum levels of TG and ALT in HFD-fed mice switched to RC. Mice were treated as described in Figure 1 and the serum levels of TG and ALT were measured. Values are expressed as means ± SEM (*n* = 8). Different letters indicate statistical significance, *P* < 0.05.

Because high serum levels of TG and ALT are the hallmarks of NAFLD and liver damage, respectively, we measured their levels and found that, despite the expected suppressive effect of the RC diet on lowering serum TG, mice given 1% DHA, but not lysine, had lower serum TG levels compared to the RC only group (Figure 2(a)). Despite the lack of a synergistic effect, combinations of DHA and lysine also decreased TG levels. ALT, however, was only reduced by 1% DHA and 1% DHA + 0.5% lysine (Figure 2(b)). These results suggest that DHA and lysine exert their beneficial effects by modulating distinct cellular targets and processes.

### 3.4. DHA and Lysine Supplemented-RC Feeding Ameliorated HFD-Induced NAFLD in Mice

**Figure 3:**
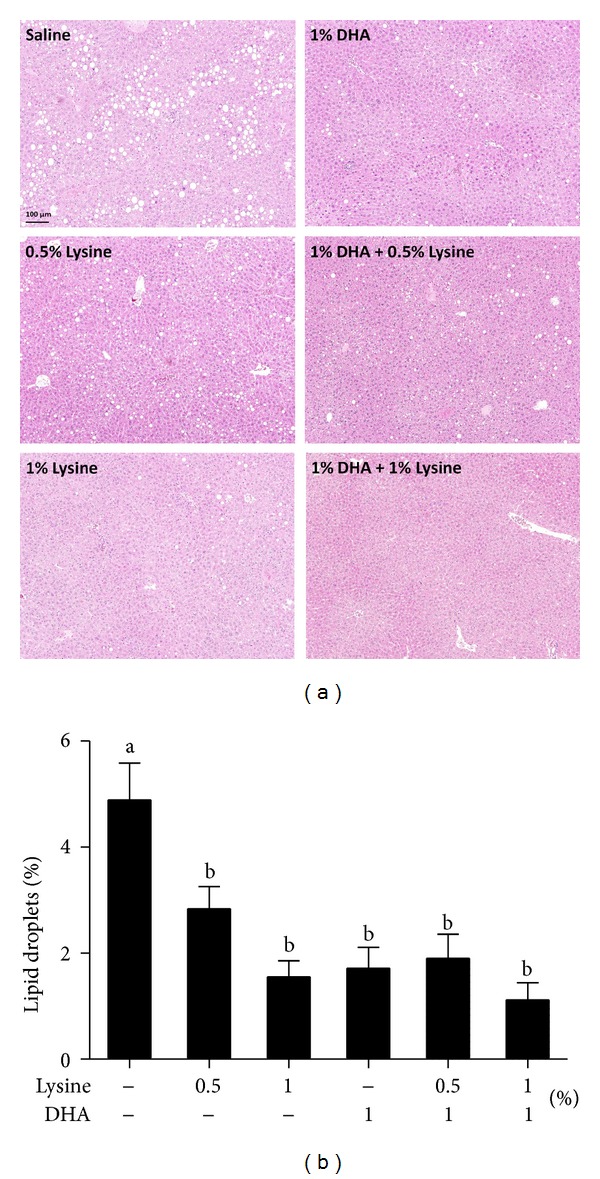
Effects of lysine and DHA supplementation on hepatic lipid droplet accumulation in HFD-fed mice switched to RC. Mice were treated as described in Figure 1. (a) Representative histology of liver sections (100x magnification with the bar indicating 100 *μ*m) stained with hematoxylin/eosin. (b) Quantification of lipid droplet area in a (*n* = 6). Data are expressed as percentage of the area of lipid droplets in the field. Different letters indicate statistical significance, *P* < 0.05.

Despite RC-induced body weight loss, histological analysis of the liver revealed that HFD-induced accumulation of lipid droplets remained apparent in the livers of saline control (Figure 3(a)) after switching to RC for 24 days. In sharp contrast, there were only a few smaller droplets in the DHA- and lysine-treated groups. Quantification by image analysis indicated that all groups supplemented with lysine, DHA, or their combinations had reduced hepatic lipid (Figure 3(b)). Histological sections of the gonadal adipose tissues revealed that, consistent with the liver data, combinations of DHA and lysine reduced the size of adipocytes compared to the RC only control without a noticeable difference among the treatment groups (Figure S2). Perhaps, adipose lipogenesis contributed to the hepatic lipid content.

### 3.5. Expression Profiles of Genes Associated with Hepatic Inflammation and Lipid Metabolism

**Figure 4:**
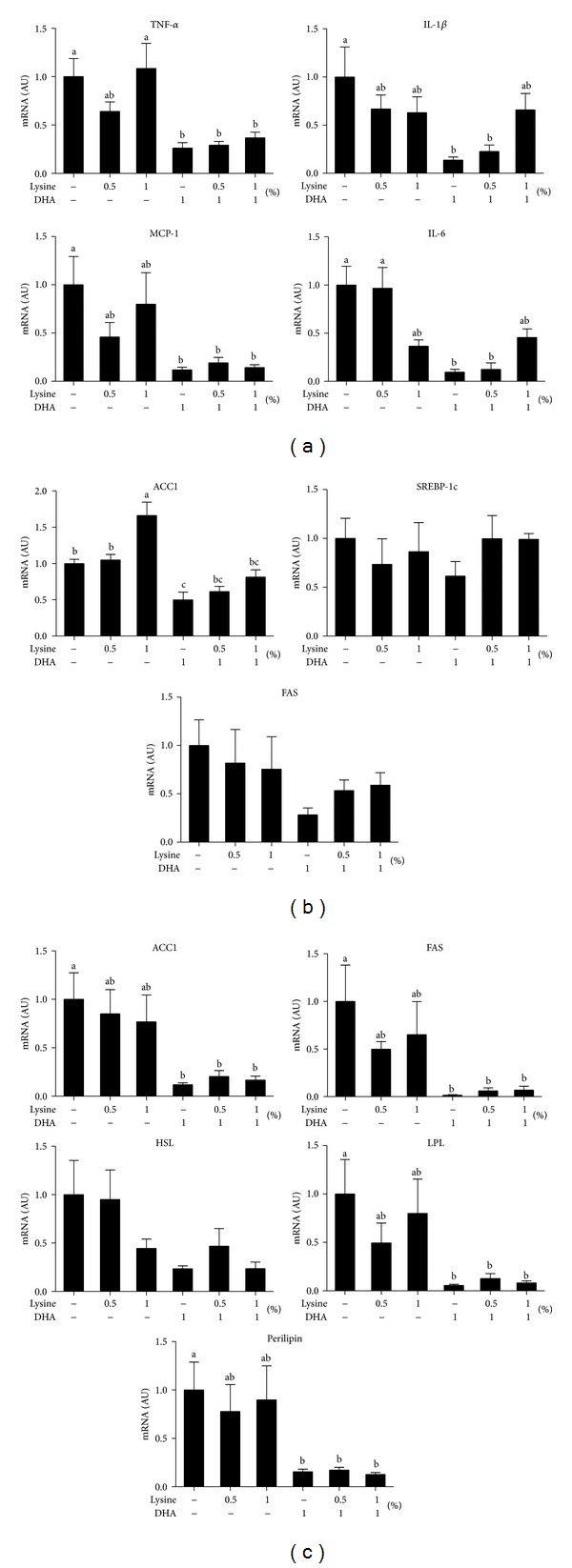
Effects of DHA and lysine supplementation on expression of hepatic proinflammatory cytokine and lipid metabolism-associated genes in HFD-fed mice switched to RC. Expression of (a) hepatic proinflammatory genes, including tumor necrosis factor-*α* (TNF-*α*), interleukin-1*β* (IL-1*β*), monocyte chemotactic protein-1 (MCP-1), and interleukin-6 (IL-6), (b) hepatic lipid metabolism-associated genes, including acetyl-CoA carboxylase1 (ACC1), sterol regulatory element-binding protein-1c (SREBP-1c), and fatty acid synthase (FAS), in the livers, and (c) gonadal adipose tissue acetyl-CoA carboxylase1 (ACC1), fatty acid synthase (FAS), hormone-sensitive lipase (HSL), lipoprotein lipase (LPL), and perilipin. Mice were treated as described in Figure 1 and the total RNA was extracted from the tissues and measured by qRT-PCR as described in Section 2. Values were normalized to *β*-actin mRNA expression in the same sample and expressed as means ± SEM (*n* = 8). Different letters indicate statistical significance, *P* < 0.05.

Because low-grade and chronic inflammation and impaired lipid metabolism contribute to NAFLD, we analyzed the effects of DHA and lysine on the expression of hepatic proinflammatory genes, including tumor necrosis factor-alpha (TNF-*α*), interleukin-1 beta (IL-1*β*), monocyte chemotactic protein-1 (MCP1), and interleukin-6 (IL-6) and lipogenic genes, including acetyl-CoA carboxylase1 (ACC1), sterol regulatory element-binding protein 1c (SREBP-1c), and fatty acid synthase (FAS). One percent DHA and 1% DHA + 0.5% lysine reduced IL-1*β*, TNF-*α*, MCP-1, and IL-6 mRNA expression (Figure 4(a)). TNF-*α* and MCP-1 expressions were also decreased by 1% DHA+ 1% lysine treatment compared to the RC only control. In sharp contrast, expression of proinflammatory cytokines was not significantly different among the saline, 0.5% lysine, and 1% lysine groups. For lipogenic genes, only 1% DHA treatment markedly diminished ACC1 mRNA expression (Figure 4(b)). These results suggest that, after weight loss, the beneficial effects of DHA on NAFLD primarily resulted from the suppression of proinflammatory genes, at least at the transcriptional level.

### 3.6. Expression Profiles of Genes Associated with Lipid Metabolism in Gonadal Adipose Tissues

Because adipose tissue plays a major role in systemic lipid and energy homeostasis and an antiadiposity effect of DHA and lysine in the gonadal adipose tissues was observed (Figure S2), the expression of lipid metabolism-associated genes was also analyzed. Supplementation with 1% DHA, 1% DHA + 0.5% lysine, and 1% DHA + 1% lysine, but not lysine alone, markedly reduced gonadal adipose tissue mRNA expression of lipogenic genes, including ACC1, FAS, lipoprotein lipase (LPL) and a lipid droplet-associated protein, and perilipin (Figure 4(c)). These results indicated that DHA supplementation, in the presence or absence of lysine, modulates adiposity and may contribute to the amelioration of hepatosteatosis.

### 3.7. DHA and Lysine Inhibit FAS and IL-6 Expressions in Human SK-HEP-1 Cells

**Figure 5:**
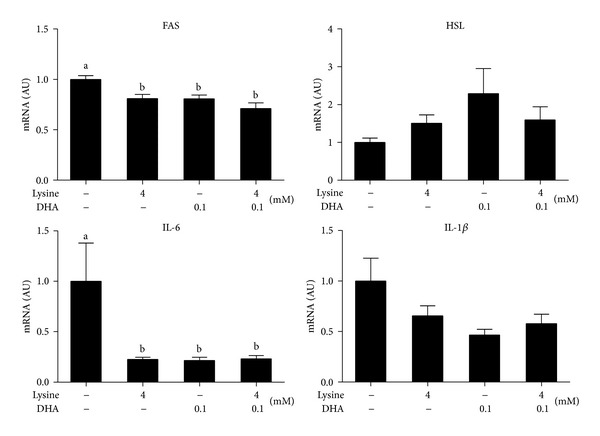
Effects of lysine and DHA treatments on the expression of lipogenic, lipolytic, and proinflammatory cytokine genes in oleic acid-induced steatotic SK-HEP-1 cells.SK-HEP-1 cells were treated with 1 mM of oleic acid for 48 h to induce steatosis and then treated with 4 mM lysine ± 0.1 mM DHA for 24 h. Total RNA samples were extracted and analyzed for the mRNA levels of fatty acid synthase (FAS), hormone-sensitive lipase (HSL), interleukin-6 (IL-6), and interleukin-1 beta (IL-1*β*) by qRT-PCR as described in Section 2. Values were normalized to *β*-actin mRNA expression in the same sample and expressed as means ± SEM (*n* = 3). Different letters indicate statistical significance, *P* < 0.05.

To verify the* in vivo* results, a human hepatocellular cell line, SK-HEP-1 cells, was challenged with 1 mM oleic acid [10] for 48 h to establish a cellular model for fatty liver disease. Then, after a 24 h supplementation of the cell culture medium with 0.1 mM DHA, 4 mM lysine, or 0.1 mM DHA + 4 mM lysine, the expression of the lipogenic gene, FAS was significantly reduced, with no effect on the lipolytic gene, HSL (Figure 5). For inflammatory genes, 0.1 mM DHA, 4 mM lysine, and 0.1 mM DHA + 4 mM lysine consistently reduced the expression of IL-6 and IL-1*β*, with the latter being of marginal statistical significance (*P* = 0.07). In summary, these results support the beneficial roles of DHA and lysine in NAFLD through modulating hepatic inflammation and lipid metabolism.

## 4. Discussion and Conclusion

NAFLD is a worldwide disease, which parallels the frequency of obesity, insulin resistance, metabolic syndrome, and type 2 diabetes [1]. Despite the recent development of therapeutic and preventive strategies [7, 8], treatments with better accessibility, convenience, cost effectiveness, and fewer side effects remain urgently in demand. In the present study, we explored such a strategy by combining the treatments of a low-fat diet, and nutrient supplementations, specifically DHA and lysine, which have separately been shown to ameliorate NAFLD. As expected, switching to RC induced body weight loss and diminishment of hepatic lipid droplet accumulation; these effects were enhanced by DHA and lysine supplementation. Of note was the more profound weight loss by lysine compared to DHA in the early stage of treatment. Moreover, the combined treatments of DHA and lysine essentially restored the hepatosteatotic condition to normal compared to the RC only group. Our current approach suggests an effective approach to treat NAFLD.

To elucidate molecular mechanisms underlying NAFLD and develop therapeutic strategies, an animal model mimicking the natural course and etiological background of this disease is essential. Consistent with previous studies [22–24], our HFD approach induced significant body weight gains (Figure S1A) with massive lipid droplet accumulation in the liver (Figure S1B) and profound hypertrophy of adipocytes (Figure S1C). In rats, 5-week HFD (70% of energy from fat) feeding increases hepatocyte lipid content without changing the body weight [25]. However, we found that 4-week-old male C57BL/6 mice fed a HFD (58.7% of energy from fat) for 23 weeks weighed significantly more than those fed the RC (low-fat chow diet) control diet (Figure S1A). Yet, similar to the rat data, no significant difference in the serum TG levels (data not shown) was observed between our HFD and RC groups. The species divergence in weight gain plus a longer interval to achieve significant hepatic lipid droplet accumulation in the mice may be attributed to species, composition of diets (59 versus 70% fat), or the duration of HFD feeding.

Since energy overload contributes to obesity and its associated metabolic diseases, including NAFLD, we reduced the energy intake by switching the diet from HFD to RC, mimicking human behavior to eat lighter when diagnosed with fatty liver disease. Switching to low-fat diet indeed reduced the body weight, as well as hepatic lipid droplet accumulation (Figures 1 and 3). Body weight gain and serum ALT activity, a marker of liver damage, are lower in rats fed diets containing 5% lysine compared to the control [19]. After 4 weeks of daily gavage with 0.5% or 1% lysine (based on average of dietary intake), 1% lysine caused additional body weight loss beginning early in the treatment period; this was not observed in other treatment groups except in the 1% lysine + 1% DHA in the latter days of treatment (Figure 1). Perhaps lysine does play a role to increase mitochondrial energy metabolism [16]. Although the slight change of dietary ingredients is not likely to affect feed consumption greatly, we cannot rule out the possibility that a part of weight loss may be due to the difference of food consumption.

Along with the enhanced body weight loss by 1% lysine alone and 1% DHA + 1% lysine (Figure 1), the serum levels of TG were consistently reduced by DHA and its combination with lysine, but not by lysine alone (Figure 2(a)), suggesting that serum TG levels are the target of DHA but not lysine action, which is consistent with a previous human study [26]. DHA, but not lysine, lowered serum ALT activity, except for the 1% lysine + 1% DHA treatment (Figure 2(b)) with a slight but nonsignificant synergistic effect for 1% DHA + 0.5% lysine group. Moreover, it appears that the higher (1%), but not lower, lysine dose neutralizes the beneficial effects of DHA on ALT activities, suggesting that the dose of lysine, when combined with DHA, may be critical in modulating beneficial effects on liver injury.

Weight loss is effective and recommended for the treatment of NAFLD [7, 8]. Indeed, in our current study, after a 4-week RC-induced weight loss, a significant amount, but not all, of the lipid droplets in the livers of HFD-fed mice was eliminated (Figure 3). Eradication of additional lipid droplets by the combined supplementations of DHA and lysine (Figure 3) suggests that DHA and/or lysine may target mechanisms distinct from those of energy homeostasis in hepatic lipid metabolism. While discrete effects of DHA and lysine were observed on body weight and serum ALT activity, DHA, lysine, and their combinations all reduced hepatic lipid (Figure 3(b)).

Supplementation with fish oil or* n*-3 PUFA decreases serum TG in both mice and humans [9, 10, 12]. Moreover, ultrasonic examination demonstrates an improvement in liver echotexture and improved hemodynamics after* n*-3 PUFA intake [12]. Mice fed a lard/safflower oil diet have significant hepatic fat droplet accumulation that is inhibited by fish-oil feeding [13]. Because we found that both DHA and lysine decreased hepatic lipid content, but only DHA reduced serum TG and ALT activity, different mechanisms are suggested for the actions of DHA and lysine.

To investigate underlying mechanisms, we analyzed the expression of selected genes associated with inflammation and lipid metabolism in the liver and gonadal adipose tissue. Chronic activation of inflammatory pathways leads to the infiltration of monocytes/macrophages into adipose tissue and liver and the subsequent activation of proinflammatory pathways and cytokine secretion by macrophages [27]. Hepatic TG accumulation may promote the expression of proinflammatory cytokines, which then induce inflammation and lead to neutrophil chemotaxis [28]. Moreover, HFD increases the expression of proinflammatory genes, such as IL-6, TNF-*α*, MCP-1, and IL-1*β*, which can be suppressed by* n*-3 PUFA treatment in mice [27, 29]. Our results also showed that RC supplemented with DHA and lysine, but not saline (RC only), suppressed the expression of hepatic proinflammatory genes (Figure 4(a)), which likely contributed to the enhanced amelioration of NAFLD. In adipose tissue, these genes were numerically, but not statistically, suppressed (data not shown).

Consistent with the reduction in hepatic lipid droplets, 1% DHA supplementation downregulated the expression of hepatic lipogenic genes, such as ACC1 and FAS, but not SREBP-1c (Figure 4(b)). ACC1 and FAS are target genes of SREBP-1c, which is the master transcription factor in hepatic lipogenesis [30]. Because posttranslational modification/processing is necessary to release the bioactive form of SREBP-1c from the ER [30] and we did not measure the transcriptional activity or the bioactive form, the lack of effect of DHA on SREBP-1c gene expression may not reflect activity of SREBP-1c as a transcription factor. For gonadal adipose tissue, DHA or its combination with lysine, but not lysine only, consistently suppressed the expression of lipogenic genes, such as ACC1, FAS, LPL, and perilipin (Figure 4(c)). This is consistent with the observation of Arai et al. [13] that fish oil exerts an antiobesity effect through the inhibition of lipid synthesis. In our mice, based on mRNA expression, the effects of DHA to modulate hepatic lipid metabolism were to suppress the inflammatory response and lipid anabolism. Although lysine was expected to decrease the serum ALT levels and the expression of proinflammatory genes, perhaps we did not observe these effects because lysine was administered at only 0.5 and 1%. Because lysine plays an essential role in the production of carnitine, a nutrient that facilitates the conversion of fatty acids into energy [31], it may contribute to the decreased hepatic lipid accumulation in our mouse study. Lastly, our* in vitro* model, for the first time, linked lysine to hepatic inflammation by showing that 4 mM lysine reduced IL-6 mRNA expression (Figure 5). The mechanism underlying lysine-modulated inflammation is worthy of further investigation.

In conclusion, we demonstrated that in combination with RC (low-fat diet), DHA treatment decreased serum TG and ALT levels and reduced hepatic lipid droplet accumulation by suppressing hepatic and adipose inflammation and restoring impaired lipid metabolism in mice previously fed HFD. Lysine also effectively reduced lipid accumulation in the liver and enhanced weight loss, but the mechanisms remain to be identified. Our current study thus provides an alternative approach to ameliorate NAFLD by supplementation of caloric-restricted diets with DHA or lysine.

## Supplementary Material

The mice were fed with HFD for 23 weeks to induce the fatty liver symptom. Two mice were randomly sacrificed from HFD and control group after 8, 12 and 16 weeks to verify fatty liver induction by histological analysis ( see Materials and Methods for details).

## Figures and Tables

**Table 1:** List of primer sequences used for real-time qPCR analysis.

m-ACC1	S 5′-TAATGGGCTGCTTCTGTGACTC-3′ A 5′-CTCAATATCGCCATCAGTCTT-3′	AY451393
m-FAS	S 5′-GGAGGTGGTGATAGCCGGTAT-3′ A 5′-TGGGTAATCCATAGAGCCCAG-3′	BC046513
m-SREBP-1c	S 5′-GGAGCCATGGATTGCACATT-3′ A 5′-GGCCCGGGAAGTCACTGT-3′	NM_011480
m-HSL	S 5′-ATGGAGCCGGCCGTGGAATC-3′ A 5′-AACGCTGAGGCTTTGATCTTGCC-3′	BC021642
m-LPL	S 5′-AGGACCCCTGAAGACAC-3′ A 5′-GGCACCCAACTCTCATA-3′	BC003305
m-*β*-Actin	S 5′-CATGTACGTAGCCATCCAGG-3′ A 5′-CTCTCAGCTGTGGTGGTGAA-3′	BC138614
m-TNF-*α*	S 5′-CCACGTCGTAGCAAACCAC-3′ A 5′-TTGTCCCTTGAAGAGAACCTG-3′	D84199
m-IL-1	S 5′-CCCTGCAGCTGGAGAGTGTGG-3′ A 5′-TGTGCTCTGCTTGTGAGGTGCT-3′	NM_008361
m-FoxO1	S 5′-CGCTTGGACTGTGACATGG-3′ A 5′-TAAATGTAGCCTGCTCACTAACTC-3′	NM_019739
m-MCP1	S 5′-CCTGTCATGCTTCTGGGCCTGC-3′ A 5′-GGGGCGTTAACTGCATCTGGCTG-3′	NM_011333
m-IL-6	S 5′-CCAGAGATACAAAGAAATGATGG-3′ A 5′-ACTCCAGAAGACCAGAGGAAAT-3′	J03783
m-Perilipin	S 5′-TCTCAGGATGAGAGCCATGA-3′ A 5′-ATGGTGTTCCGGAGAGTGTT-3′	AY161165
h-FAS	S 5′-ACAGGGACAACCTGGAGTTC-3′ A 5′-CTGTGGTCCCACTTGATGAGT-3′	NM_004104.4
h-HSL	S 5′-TCAGTGTCTAGGTCAGACTGG-3′ A 5′-AGGCTTCTGTTGGGTATTGGA-3′	NM_005357.2
h-IL-1	S 5′-GGGCCTCAAGGAAAAGAATC-3′ A 5′-TTCTGCTTGAGAGGTGCTGA-3′	NM_000576
h-IL-6	S 5′-TGGAGATGTCTGAGGCTCATTCT-3′ A 5′-GAAGGCAACTGGACCGAAGG-3′	NM_000600
h-*β*-Actin	S 5′-GAAGATCAAGATCATTGCTCCTC-3′ A 5′-CTAAGTCATAGTCCGCCTAGAAG-3′	NM_001101

## Abbreviations

ACC1: Acetyl-CoA carboxylase 1
ALT: Serum alanine transaminase
ANOVA: One-way analysis of variance
Ct: Threshold cycle
DHA: Docosahexaenoic acid
DMEM: Dulbecco's Modified Eagle Medium
FAS: Fatty acid synthase
FLD: Fatty liver disease
HFD: High-fat diet
HSL: Hormone-sensitive lipase
IL-1*β*: Interleukin-1 beta
IL-6: Interleukin-6
LPL: Lipoprotein lipase
MCP1: Monocyte chemotactic protein-1
NAFLD: Nonalcoholic FLD
PUFA: Polyunsaturated fatty acid
RC: Regular chow
SREBP-1c: Sterol regulatory element-binding protein 1c
TG: Triglyceride
TNF-*α*: Tumor necrosis factor-alpha.
