# Supplementary figures and images for: Enhanced Amelioration of High-Fat Diet-Induced Fatty Liver by Docosahexaenoic Acid and Lysine Supplementations
Source: Biomed Res Int. 2014 May 25;2014:310981. doi: 10.1155/2014/310981 (PMC4055637; doi:10.1155/2014/310981)

(a)

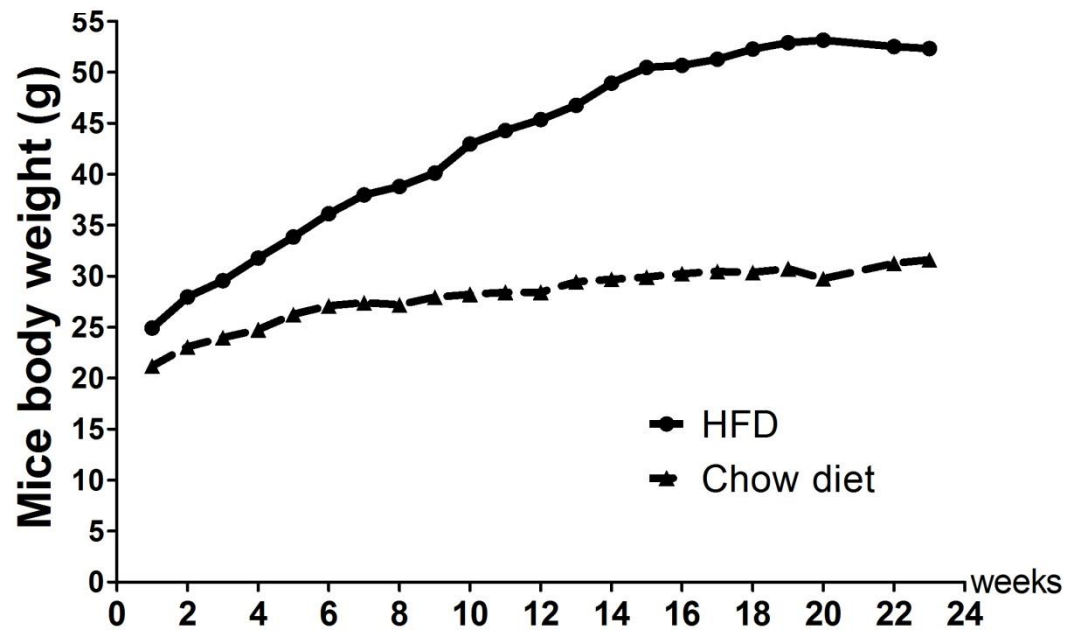

(b)

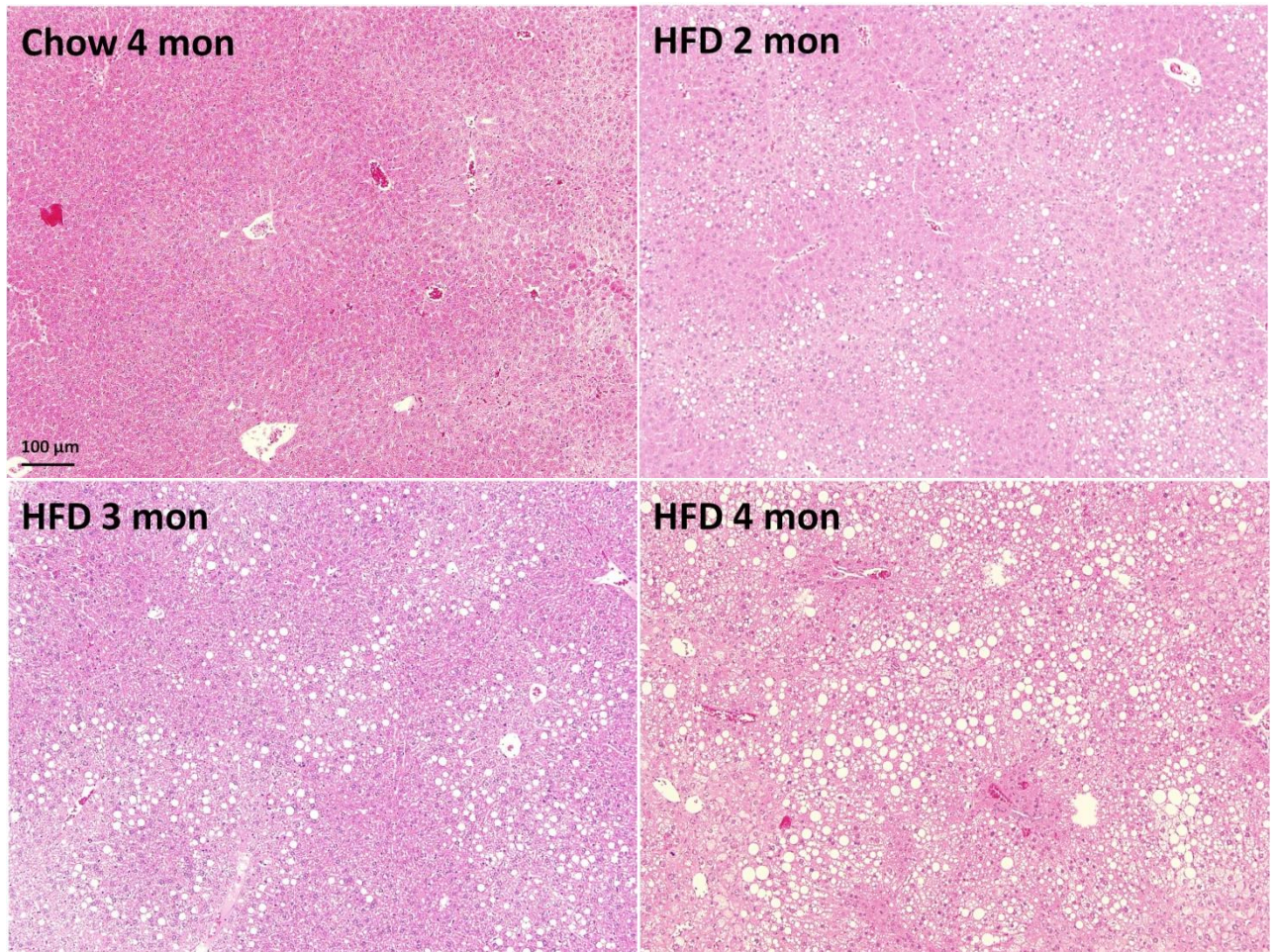

(c)

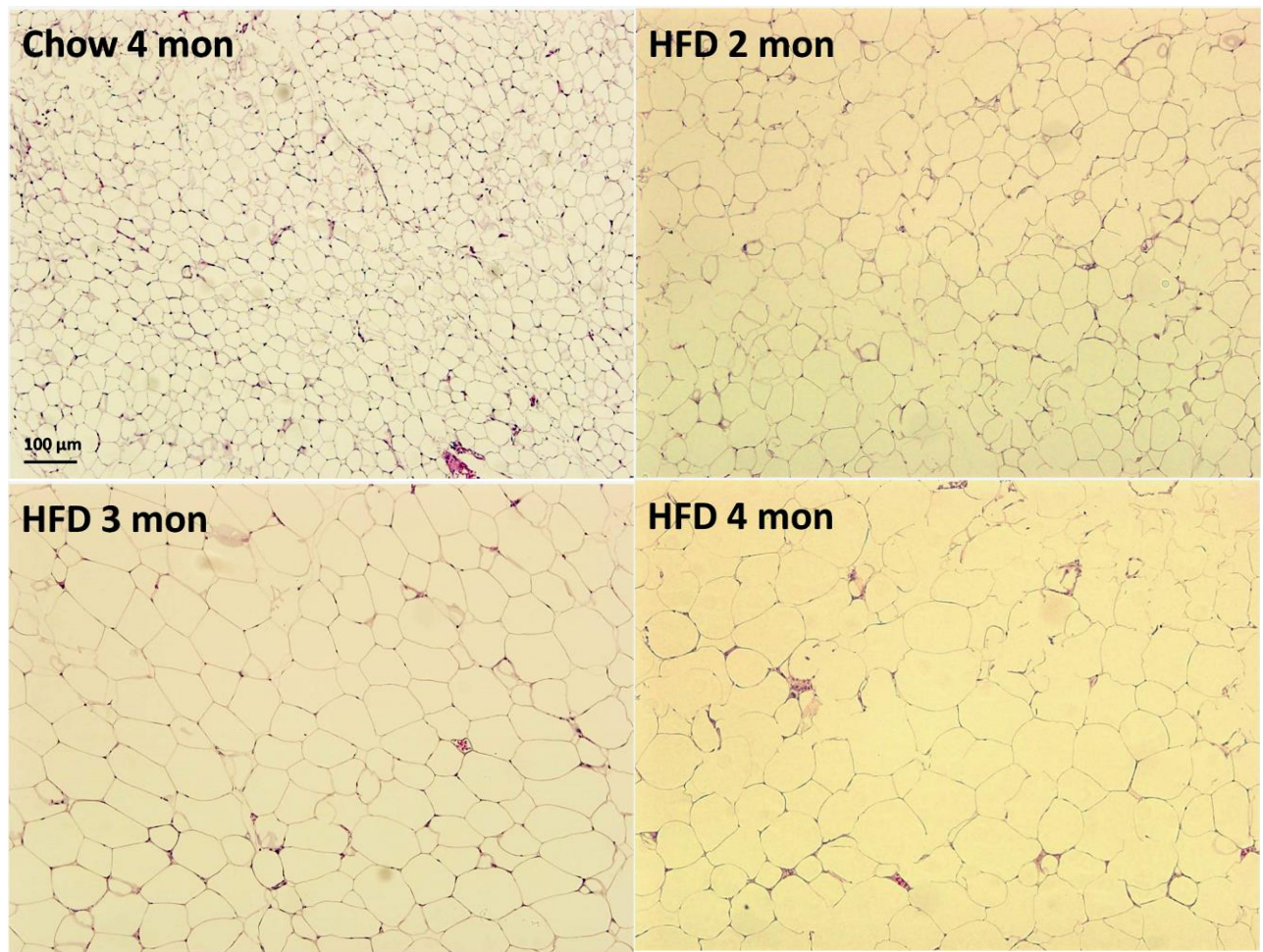

Supplement: Supplementary file 1 — The mice were fed with HFD for 23 weeks to induce the fatty liver symptom. Two mice were randomly sacrificed from HFD and control group after 8, 12 and 16 weeks to verify fatty liver induction by histological analysis ( see Materials and Methods for details). [file 310981.f1.zip › mat.310981.v3/310981.suppl fig1 .pdf]

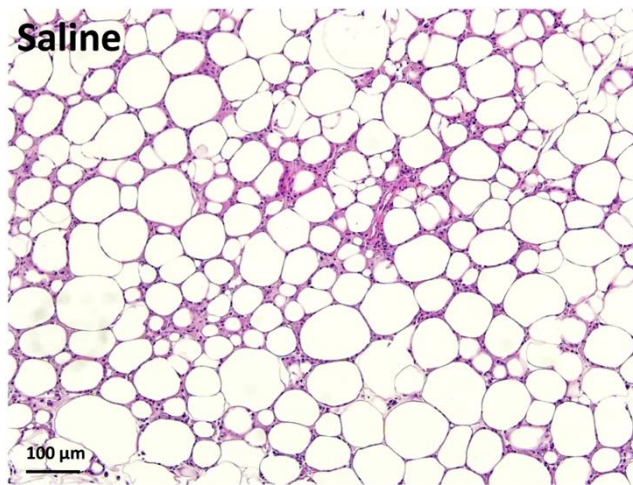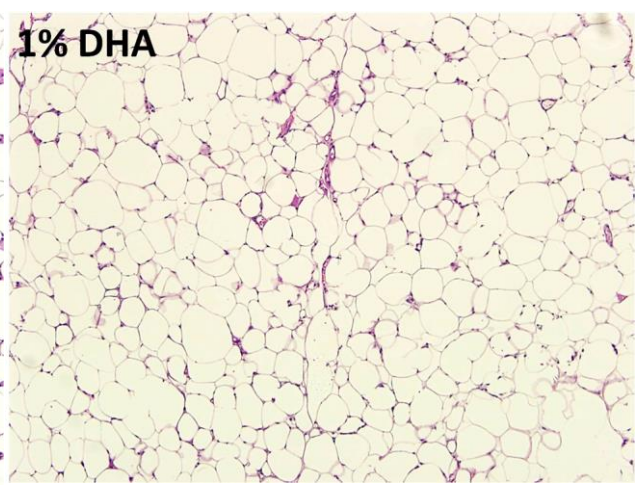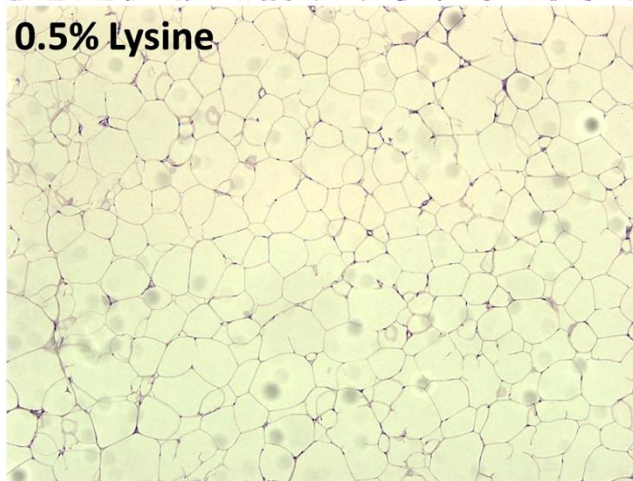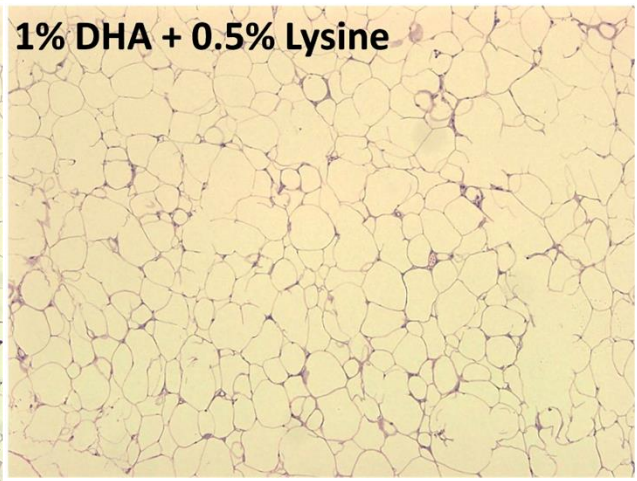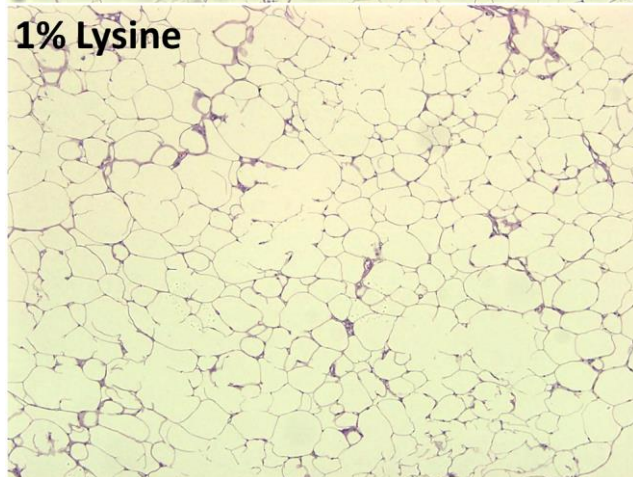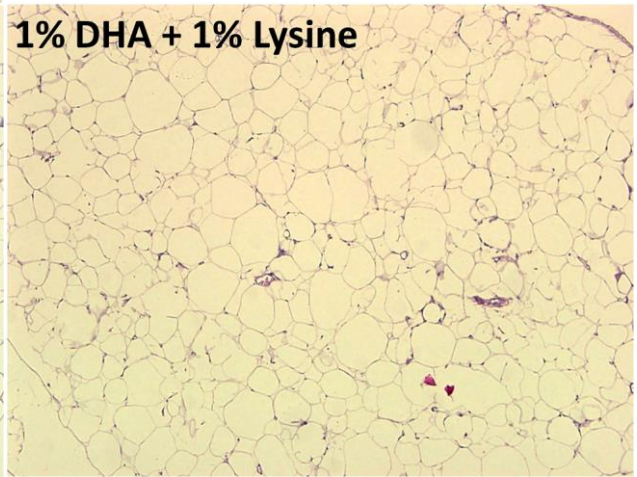

Supplement: Supplementary file 1 — The mice were fed with HFD for 23 weeks to induce the fatty liver symptom. Two mice were randomly sacrificed from HFD and control group after 8, 12 and 16 weeks to verify fatty liver induction by histological analysis ( see Materials and Methods for details). [file 310981.f1.zip › mat.310981.v3/310981.suppl fig2 .pdf]
